# The Efficacy of Botulinum Toxin A Injection in Pelvic Floor Muscles in Chronic Pelvic Pain Patients: A Double‐Blinded Randomised Controlled Trial

**DOI:** 10.1111/1471-0528.17991

**Published:** 2024-11-13

**Authors:** Melle A. Spruijt, Wenche M. Klerkx, Kim Notten, Hugo van Eijndhoven, Leonie Speksnijder, Manon H. Kerkhof, Kirsten B. Kluivers

**Affiliations:** ^1^ Department of Obstetrics and Gynaecology Radboud University Medical Center Nijmegen The Netherlands; ^2^ Department of Obstetrics and Gynaecology St. Antonius Hospital Nieuwegein The Netherlands; ^3^ Department of Obstetrics and Gynaecology Isala Hospital Zwolle The Netherlands; ^4^ Department of Obstetrics and Gynaecology Amphia Hospital Breda The Netherlands; ^5^ Department of Gynaecology and Reconstructive Pelvic Surgery Curilion Women's Health Clinic Haarlem The Netherlands

**Keywords:** botulinum toxin A, chronic pain, pelvic floor muscle, pelvic pain, physical therapy

## Abstract

**Objective:**

To evaluate and compare the efficacy and safety of Botulinum Toxin A (BTA) injections versus placebo injections, combined with pelvic floor muscle therapy (PFMT), in women with chronic pelvic pain (CPP).

**Design:**

Randomised, double‐blinded clinical trial (January 2020–April 2023).

**Setting:**

This multicentre study was conducted at four hospitals in the Netherlands.

**Population and Sample:**

Ninety‐four women with CPP and increased pelvic floor muscle tone despite previous PFMT, were enrolled.

**Methods:**

Participants received either BTA injections (100 units) or placebo injections into the pelvic floor muscle, followed by four PFMT sessions.

**Main Outcomes and Measures:**

Primary outcomes included the number of women with at least a 33% reduction in pain and those reporting (very) much improvement of their pain. Secondary outcomes covered quality of life and pelvic floor function. Follow‐up visits were scheduled at 4, 8, 12, and 26 weeks post‐treatment. Mixed models for repeated measurements were used for analysis.

**Results:**

A 33% reduction or more in average pain score was reported by 15 participants (33%) after BTA treatment and 9 participants (20%) after placebo treatment (odd ratio placebo/BTA 1.88; 95% CI 0.72–4.90, *p* = 0.19). In both groups, 8 women (17%) reported their improvement as (very) much better (odd ratio placebo/BTA 0.947; 95% CI 0.32–2.80, *p* = 0.92). Pelvic floor resting activity decreased significantly after BTA treatment compared to placebo (*p* = 0.001).

**Conclusion:**

The results from this study do not support the use of BTA injections in the management of CPP in women.

## 1 Introduction

Chronic pelvic pain (CPP) in women is an important healthcare concern [1]. It is estimated that 6%–27% of women worldwide suffer from CPP, leading to a substantial impact on quality of life, increased healthcare costs, and productivity loss [1, 2]. CPP is a multifactorial condition and therefore often not recognised and not treated sufficiently [3].

Increased muscle tension can be both a cause and a consequence of chronic pain [4]. As a reaction to chronic pain, muscles in the affected area can remain in a contracted state as a counterproductive protective mechanism. Chronic muscle contraction is associated with a disproportionate release of acetylcholine and other neurogenic inflammatory substances (substance P, calcitonin gene‐related peptide and glutamate). Together these neuropeptides activate a complex cascade resulting in a direct stimulation of peripheral nociceptors, and lowering of the threshold for pain nociception. Signals proceed to the central nervous system leading to sensitisation and maintenance of the pain sensation, even after the primary cause of pain nociception has vanished [5, 6, 7]. In addition, prolonged and increased muscle tension can lead to reduced blood flow, tissue ischemia, and the accumulation of metabolic waste products, further exacerbating pain nociception, and leading to a cycle of pain and chronic muscle contraction [8, 9].

Pelvic floor muscle therapy (PFMT) is widely used in the treatment of CPP, but there is no high‐grade evidence for its efficacy [10]. When PFMT fails, a multidisciplinary approach is advised, and a more invasive intervention may be proposed [11]. BTA injections in the pelvic floor muscles are one of the options. BTA injections have been described in the treatment of abnormal inappropriate muscle contraction in other disciplines [12]. By inhibiting the release of acetylcholine, BTA produces a localised, partial, and reversible chemical denervation of muscle, which results in localised muscle weakness or temporarily paralysis [13]. BTA may block a vicious circle of pain‐contraction‐more pain. Moreover, BTA reduces central sensitisation by blocking glutamate and substance P [5]. Total recovery of muscle strength can be expected after 3–6 months [12].

There is limited long‐term evidence regarding the efficacy of BTA as a treatment for women with CPP [14]. In this randomised controlled trial, our objective was to compare the efficacy of BTA injections versus placebo injections, in conjunction with PFMT, among women with CPP. We hypothesize that women with CPP receiving BTA injections in combination with PFMT will experience greater reductions in pain and improvements in quality of life compared to those receiving placebo injections with PFMT.

## 2 Patients and Methods

### 2.1 Study Design

This investigator‐initiated multicentre randomised, double‐blind, placebo‐controlled trial was conducted in four Dutch hospital clinics (university, *n* = 1; teaching *n* = 3) from January 2020 to April 2023. This study was approved by the medical ethics committee of Arnhem‐Nijmegen (ID: 2017–3130) and all local ethical committees in accordance with the Declaration of Helsinki.

Details of our study protocol are published [15] and the trial was registered at the European Union Drug Regulating Authorities Clinical Trials Database (2017‐001296‐23).

### 2.2 Participants and Recruitment

Women eligible for the study reported CPP according to ICS criteria [1] combined with increased muscle tension despite PFMT by a registered therapist. Inclusion criteria were a minimal age of 16 years and the ability to undergo vaginal examination. Exclusion criteria were pregnancy/lactation, prior pelvic floor BTA treatment, or hypersensitivity to BTA.

Written informed consent was obtained from all participants. Ethnicity data were gathered through investigator observation and, when uncertain, participants were asked to select from predetermined ethnicity categories. These details were collected to enhance readers' understanding of the generalisability of outcomes.

### 2.3 Intervention and Data Collection

All eligible patients presenting at the four study sites were asked to participate. Patients were also informed about the study by pelvic floor physical therapists, gynaecologists from other national hospitals and a Dutch patient foundation. In case of eligibility, they were referred to one of the study sites.

Prior to the treatment, participants were asked to complete the web‐based questionnaires, detailed below. Also, participants underwent a pelvic floor evaluation by a standardised PFMT protocol, which included the use of an EMG device (Multiple Array Probe Leiden, MAPLe [16]). These evaluations were conducted by registered pelvic floor physical therapist at baseline and at all future points in time. The MAPLe provides EMG‐registration (measurement) and biofeedback (treatment) on 24 different muscle regions of the pelvic floor. Six registered physical therapists conducted the measurements and treatment.

### 2.4 Randomisation

Participants were randomly assigned to receive either a BTA injection or placebo injection in equal patient numbers. The 94 sealed opaque envelopes, containing the treatment assignments, were shuffled and randomly distributed over the study sites. Per study site, an independent health care professional was responsible for randomisation on the day of treatment and for the preparation of the investigation product.

BTA was securely stored in the clinical pharmacy and collected on treatment day by an independent healthcare professional. The healthcare professional prepared the medication in a separate and closed room. Either 100 units of BTA (Botulinum Toxin A, Allergan/Abbvie) were dissolved in a syringe with 6 mL of saline (0.9% NaCl) or a syringe containing only 6 mL of saline (0.9% NaCl) was prepared. Both BTA and NaCl are colourless. The participant, physical therapist, and gynaecologist were blinded to the nature of the treatment arm. Six specialised urogynaecologists (maximum 2 per study site) administered the injections.

The participant was placed in dorsal lithotomy position. The pelvic floor muscle was located by digital vaginal palpation. The vaginal mucosa was pierced with an Iowa trumpet needle proximal to the hymenal remnants. The direction was posterolateral, and the depth was 10 mm into the m. puborectalis and/or m. pubococcygeus left and right. The syringes were emptied at the sites of increased muscle tension at digital palpation (2–6 sites with a maximum of 3 sites per side, 1–3 mL per site). Injections sites were checked for haemostasis.

Participants received PFMT 4, 8, 12, and 26 weeks after treatment with concurrent questionnaires. At all visits, a physical therapist performed a standardised clinical examination, evaluation with MAPLe device and provided PFMT. The main treatment focused on educating patients on pelvic floor muscles through biofeedback‐guided exercises, with the physical therapist personalising treatment for each participant's needs.

### 2.5 Outcomes

The primary outcome of this study was a minimum of 33% reduction in pelvic pain using the average pain score of the past 4 weeks ranging from 0 to 10 (painDETECT [17]), and the Patient Global Impression of Improvement (PGI‐I) reporting “much better” or “very much better” at the 26‐week mark following the injection [18, 19]. Two outcome measures were chosen because the lack of a standardised outcome measure in chronic pain research. A pain score reduction and impression of improvement were thought both to be equally relevant as outcomes in chronic pain patients.

Secondary outcomes included current, minimum, and maximum pain intensity (VAS scores ranging from 0 to 10 and assessing the pain at the moment of completion of the questionnaire), and the following questionnaires: pelvic floor distress inventory (PFDI‐20) [20], pelvic floor impact questionnaire (PFIQ‐7) [20], euroQol‐ 5 Dimension (EQ‐5D) [21], painDETECT, pain catastrophizing scale (PCS) [22], hospital anxiety and depression scale (HADS) [23] and the pelvic organ prolapse/incontinence sexual questionnaire‐IUGA revised (PISQ‐IR) [24]. Another secondary outcome was examining the pelvic floor muscles and their activity using the MAPLe device.

### 2.6 Sample Size

With an 80% statistical power and an assumed two‐sided alpha level of 0.05, the study aimed to detect a 33% reduction in average pain score in the intervention group versus placebo. Based on datasets provided by Lewis et al. [25] and Johnson et al. [26], the ANCOVA procedure as outlined by Brom (2007) established that a sample size of 94 individuals (47 per group) was necessary for this trial.

### 2.7 Statistical Analysis

Statistical analysis was conducted based on the intention‐to‐treat principle as outlined in the pre‐specified statistical analysis plan (Supplement 2). The primary endpoints were analysed using Chi‐square tests. In addition, ‘mixed models’ for repeated measurements in combination was used to assess the changes in primary and secondary outcomes over time. Missing data were assumed to occur randomly and managed using mixed models analysis. The analyses were performed with IBM SPSS version 29.0 and RStudio version 2023.06.2.

Subgroup analyses were planned to investigate the possible effect on the primary outcome of treatment of the following pre‐defined interaction terms: menopausal status, sexual activity, negative sexual experience, HADS depression scale at baseline, previous operations, parity, duration of complaints and use of opioids.

## 3 Results

**FIGURE 1:**
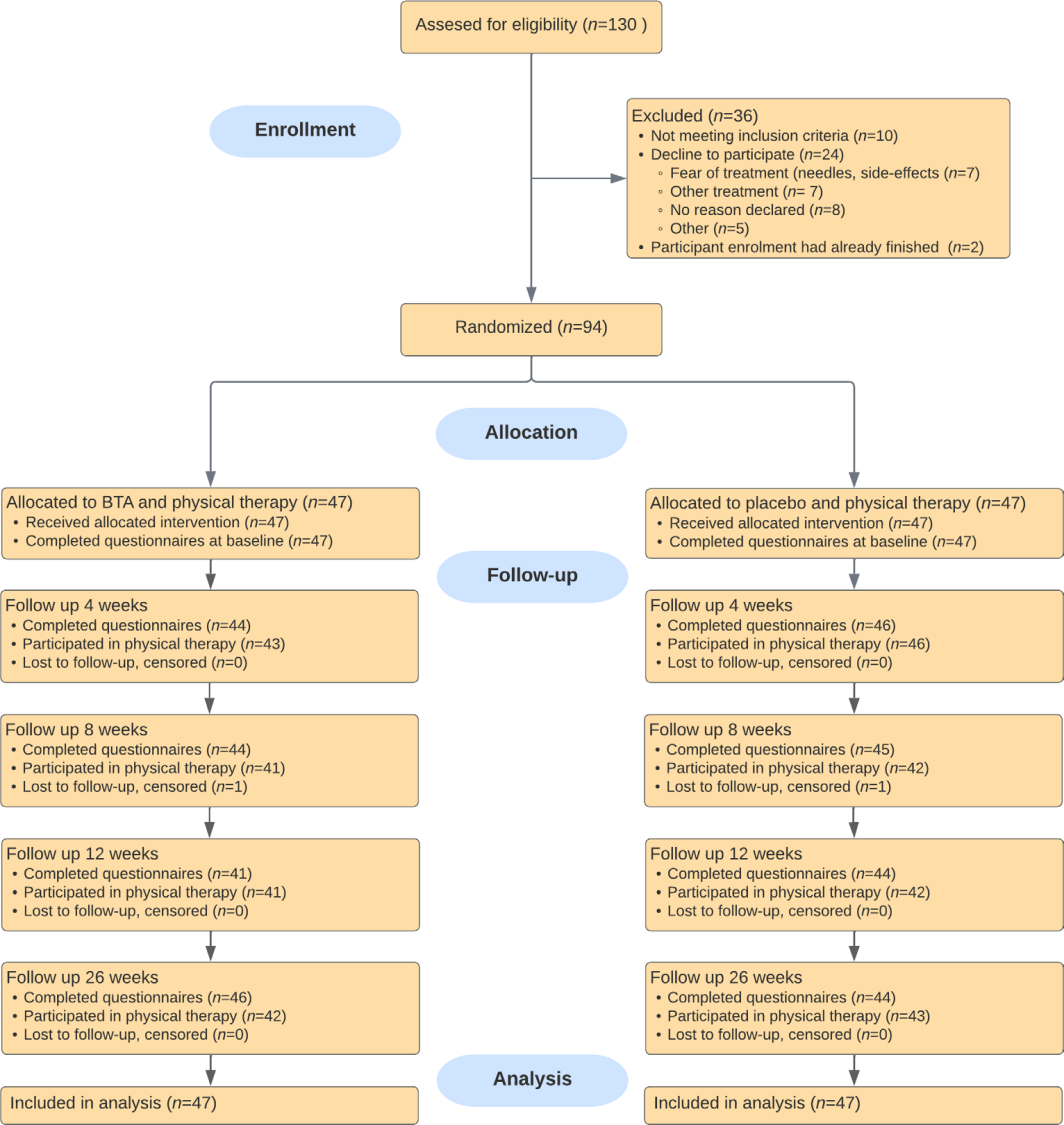
Participant flow.

In total, 94 women were randomised and the flowchart of inclusions is shown in Figure 1. Baseline characteristics showed no statistically significant differences between the treatment groups (Table 1). Two participants were lost to follow‐up due to COVID‐19 (one in each group).

**TABLE 1:** Baseline characteristics by treatment group.

Characteristics	BTA (*n* = 47)	Placebo (*n* = 47)
Age, median [IQR], years	47 [33–61]	45 [33–65]
Ethnicity: Caucasian (%)	46 (98)	46 (98)
Body mass index (BMI, kg/m^2^), median [IQR]	25 [22–27] ^y^	25[23–29] ^Ω^
Parity, No. (%)		
Nulliparous	12 (25)	12 (25)
Primiparous	13 (28)	13 (28)
Multiparous	22 (47)	22 (47)
Postmenopausal, No. (%)	20 (43)	20 (43)
Relationship status: in a relationship, No. (%)	39 (87)^Ω^	36 (77)
Sexually active, No. (%)	29 (62)	24 (51)
History of sexual abuse, No. (%)	8 (18)^Ω^	8 (18)^Ω^
Smoking, No. (%)	5 (11)	4 (9)^y^
Surgical history, No. (%)		
Abdominal surgery	1 (2)	4 (9)
Laparoscopic surgery	7 (15)	8 (17)
Prolapse surgery	8 (17)	5 (11)
Other	17 (36)	17 (36)
Average pain score^a^, mean [SD]	6.4 [0.3]^Ω^	6.5 [5.9–7.1]^Ω^
Duration of CPP, No. (%)		
1 or 2 years	12 (27)^Ω^	12 (26)^y^
3 years or longer	33 (73)	34 (74)
Use of pain medication, No. (%)		
No use of pain medication/ paracetamol	13 (28)	19 (40)
Non‐steroidal anti‐inflammatory drugs (NSAIDs)	10 (21)	11 (23)
Neuropathic pain medication^b^	17 (36)	17 (36)
Opioids	4 (9)	4 (9)
Other chronic pain syndromes (not CPP)^c^, No. (%)	6 (13)	9 (20)

*Note:* Data presented as number (percentage), mean (standard deviation) or as median (interquartile range) as appropriate.

Abbreviations: %, percentage; BTA, Botulinum toxin A; CP*P*, chronic pelvic pain; IQR, interquartile range; No, number of participants; SD, standard deviation.

^a^ Average pain score past 4 weeks, scale 0–10.

^b^ Amitriptyline, gabapentin, pregabalin and nortriptyline.

^c^ Including: fibromyalgia, piriformis syndrome, irritated bowel syndrome, interstitial cystitis, and provoked vulvodynia.

^Ω^ Data from two participants are missing.

^y^ Data from one participant is missing.

During the 26 weeks follow‐up, one participant was unblinded due to health reasons, which occurred after the final PFMT session but before the completion of the last questionnaire.

During the trial, compliance with the prescribed PFMT was similar in both groups, 37 participants (79%) of participants in the intervention group and 38 participants (81%) in the placebo group successfully completing all four scheduled physical therapy sessions. The primary factor leading to non‐compliance was attributed to COVID‐19 or personal medical conditions.

The primary endpoint of a minimum of 33% reduction in average pain score was attained by 15 participants (33%) in the BTA group and 9 participants (20%) in the placebo group at the 26‐week follow‐up (odd ratio placebo/BTA 1.88; 95% CI 0.72 to 4.90; *p* = 0.19). In both treatment groups, 8 women (17%) reported their improvement as either “much better” or “very much better” (odd ratio placebo/BTA 0.947; 95% CI 0.32 to 2.80, *p* = 0.92, Table 2).

**TABLE 2:** Primary outcome of treatment success: Average pain score past 4 weeks and patient global impression of improvement.

Outcomes	BTA		Placebo		*p* *
Average pain score, past 4 weeks^a^		No./total		No./total	
Baseline	6.4 [5.8–7.0]	47/47	6.5 [5.9–7.1]	47/47	
4 weeks follow‐up	5.6 [5.0–6.2]	44/47	6.1 [5.5–6.7]	46/47	
8 weeks follow‐up	5.1 [4.5–5.7]	44/47	5.9 [5.3–6.5]	45/47	
12 weeks follow‐up	5.2 [4.6–5.8]	41/47	5.8 [5.1–6.7]	44/47	
26 weeks follow‐up	5.1 [4.6–5.7]	46/47	5.9 [5.2–6.5]	44/47	
					0.51**
Number of participants with minimum 33% reduction in pain score	15 (33%)	46/47	9 (20%)	44/47	0.19
PGI‐I^b^		No./total		No./total	
4 weeks follow‐up	4 (9%)	44/47	1 (2%)	46/47	
8 weeks follow‐up	11 (25%)	44/47	3 (7%)	45/47	
12 weeks follow‐up	9 (22%)	41/47	4 (9%)	44/47	
26 weeks follow‐up	8 (17%)	46/47	8 (18%)	44/47	
					0.05***
Number of participants with PGI‐I (very) much improved at 26 weeks	8 (17%)	46/47	8 (18%)	44/47	0.92

*Note:* Data are presented as mean (95% confidence interval [CI]) or as number (percentage).

Abbbreviations: BTA, Botulinum toxin A; No./total, number of participants competed questionnaire/total number of participants in treatment group; PGI‐I, patient global impression of improvement.

^a^ Pain score: average pain score last 4 weeks, scale 0–10.

^b^ PGI‐I 1 or 2: “much better” or “very much better”.

* Test for statistical significance BTA versus placebo.

** Based on linear mixed models and ANOVA.

*** Based on linear mixed models and ANOVA, PGI‐I 1–7.

### 3.1 Secondary Outcomes

#### 3.1.1 Pain Scores and PGI‐I Over Time

**FIGURE 2:**
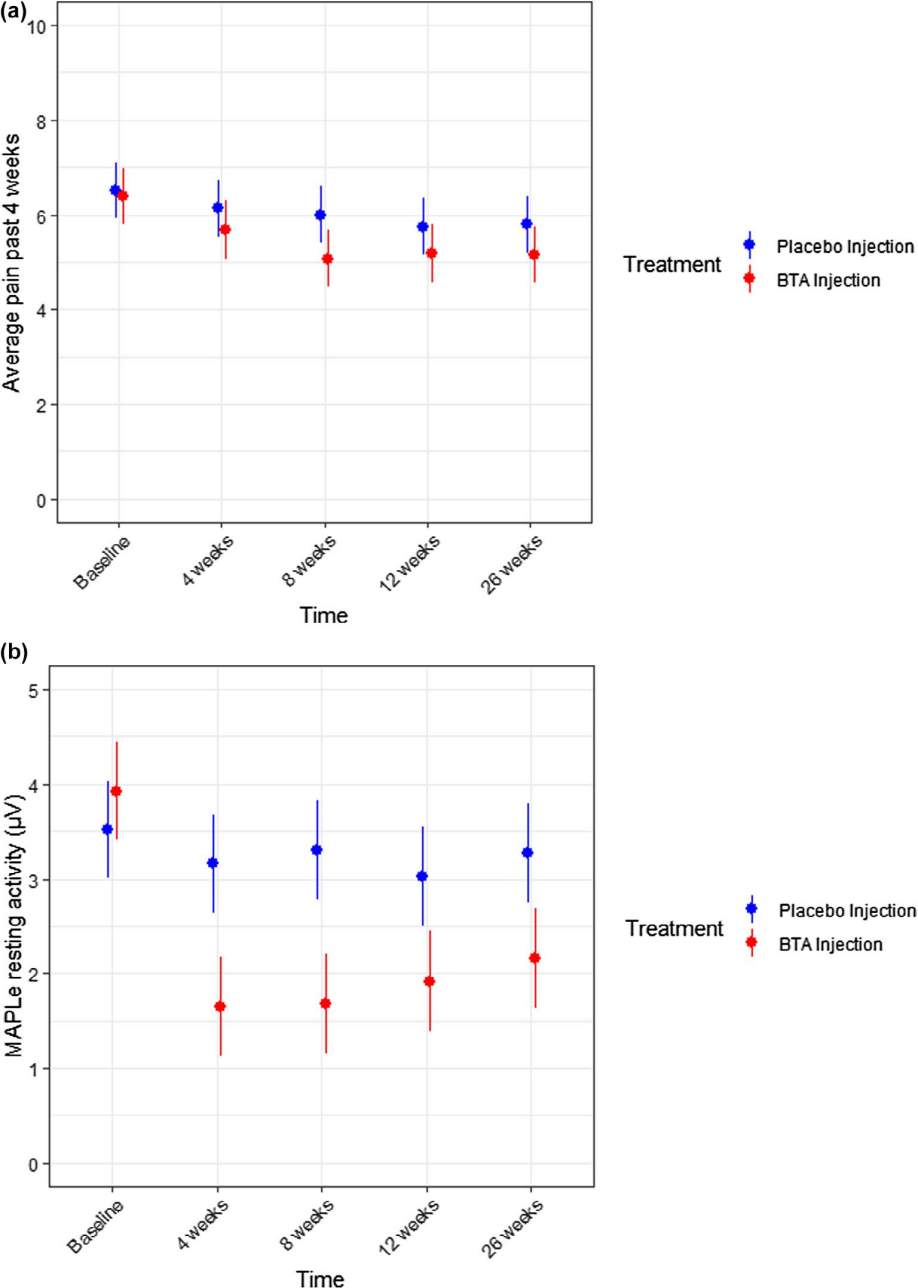
(a) Average pain score past 4 weeks; (b) Pelvic floor resting activity measure by MAPLe (μV).

Independent of the treatment group, a significant improvement in average pain score was observed at 12 (−0.7 points; 95% CI −1.3 to −0.2) and 26 weeks (−0.7 points; 95% CI −1.2 to −0.07) after treatment (Figure 2a). Current, minimum and maximum VAS pain scores are shown in Table S1.

At the 8‐week follow‐up, a significant difference was observed between groups, with 11 participants (25%) reporting to feel either “much better” or “very much better” after BTA treatment and 3 participants (7%) after placebo treatment (risk estimate odds ratio (OR) BTA treatment 0.56; 95% CI 0.39 to 0.81). However, over time, no difference was seen between BTA and placebo in PGI‐I scores (*p* = 0.05).

#### 3.1.2 MAPLe Outcomes

Following BTA treatment, a notable decrease of the Pelvic Floor Resting Activity was observed across all measured time points, as illustrated in Figure 2b (BTA vs. placebo *p* ≤ 0.001).

#### 3.1.3 Quality of Life

Table S1 shows a significant improvement in pelvic floor function (26 weeks total scores: PFIQ‐7 −24.7; 95% CI −38.9 to −10.6; PFDI‐20 −11.6; 95% CI −3.9 to −19.3) and pain catastrophizing (26 weeks: PCS total score −5.6; 95% CI −3.2 to −8.0; maximum VAS score −0.7; 95% CI −0.3 to −1.2) at every time point compared to baseline in both groups. However, these changes were independent of intervention. At 26 weeks post treatment an improvement in HADS depression scale (−1.0; 95% CI −0.2 to −1.7) and EQ5D health scale (8.9; 95% CI 3.4 to 14.4) was seen, independent of treatment arm. There was a significant cross over in the sexually (in)activity throughout the study in both groups. The overall sexual activity rates were similar between groups and remained relatively stable throughout the study (approximately 51%–62%).

#### 3.1.4 Subgroups

There was no variation in treatment effect in predefined subgroups. No conclusions could be drawn from the subgroup analysis on opioid use, due to small sample sizes within these groups. There were no other associations observed between pre‐specified patient characteristics and effect of treatment on average pain.

### 3.2 Safety

Immediate post‐treatment bleeding, resolved by 5–10 min of tamponade occurred in 2 participants (4%) in both treatment groups. There were no allergic reactions reported, and no other immediate side effects post‐injection. In the first week following treatment, 4 participants (8%) of the BTA group and 1 participant (2%) of the placebo group reported mild bleeding without need for treatment (*p* = 0.053). Stress urinary incontinence was observed in 1 participant after BTA treatment (*p* = 0.212), while no cases of faecal incontinence or infection were reported. Constipation was reported by 3 participants (6%) following BTA treatment, whereas none after placebo (*p* = 0.08).

### 3.3 Protocol Violations and Adverse Events

As outlined above, 19 out of 94 patients (20%) had cancelled one or more PFMT sessions. Throughout the study period, 1 participant conceived resulting in a protocol violation. The pregnancy and delivery proceeded without complications. One adverse event might be associated with the study: a participant in the BTA group, previously diagnosed with mental health issues, experienced severe distress and intense pain 15 weeks after the injection. The other adverse event, which was unlikely related to the study, involved an Anterior Cutaneous Nerve Entrapment Syndrome (ACNES) operation in week 4 in the BTA group.

## 4 Discussion

This multicentre randomised, double‐blind, placebo‐controlled trial showed no significant difference in efficacy between BTA injections and placebo injections in women with CPP. Both groups exhibited similar changes 6 months after the injection in their average pain score over the last 4 weeks. Although the PGI‐I was better after BTA treatment at 8 weeks follow‐up, this difference was not sustained at 26 weeks follow‐up. Interestingly, a statistically significant decrease in the pelvic floor resting activity was observed following BTA treatment but this decrease did not correspond to a reduction in pain scores.

A systematic review and meta‐analysis has reported improvement after BTA injections regarding non‐menstrual pelvic pain, dyspareunia, and pelvic floor resting activity [28]. The 2 RCT's [14, 27] included in the meta‐analysis did not find a difference in pain between BTA and placebo treatment. The most recent RCT by Dessie et al. [27] evaluated the effect of 200 U onabotulinum injected into the pelvic floor muscles, in combination with weekly PFMT, compared to placebo in women experiencing myofascial pelvic pain. In that study, 59 participants received 20 injections of 1 mL distributed bilaterally at the locations of patient‐reported pain in the muscles. Similar to our findings, Dessie et al. reported that BTA injections were not effective compared to placebo injections in reducing muscle pain, as measured with a VAS score at 12 weeks follow‐up. Despite differences in inclusion criteria, sample size, BTA dosage, number of injection sites, follow‐up duration and statistical methods, our results align with those of Dessie et al. In the observational studies included in the meta‐analysis, efficacy of BTA injections was shown to some extent. However, it was concluded that the improvement in symptoms may have been attributed to a placebo effect [29], effect of wet‐ and/or dry needling [30, 31] and/or PFMT [11]. Importantly, safety concerns were not identified in the present RCT or in previous studies.

The average pain score over the past 4 weeks from the painDETECT questionnaire was chosen as our primary outcome. This was a deliberate choice, aimed at a comprehensive assessment of CPP severity [32]. This score is not influenced by the specific moment of questionnaire completion, whether at rest or post‐mobilisation, such as in our current, minimum and maximum score. However, it is worth noting that the VAS current pain score may offer greater reliability owing to its immediacy and simplicity. In case we had chosen another primary outcome (current pain score), the conclusions would have been similar.

A significant reduction in pelvic floor resting activity, as measured with MAPLe, was seen after BTA injection in the present RCT. Previous studies have also documented lower muscle tones [14, 33] without a concomitant reduction in pain. In chronic migraine patients, however, multiple onabotulinum injections into the head and neck were superior to placebo injections in reducing headache frequency. This study suggested that muscle relaxation contributed to pain relief [34]. These findings were not replicated in the present study and underline the complexity of the pathophysiology of CPP.

It is questionable whether the changes over time that we observed within both groups are clinically relevant. The combination of injections into the pelvic floor muscles (BTA or NaCl) and PFMT, along with 6 months' time, resulted in an improvement in the VAS average pain score of 0.8 points (95% CI −1.36 to −0.54) representing 12% reduction and only 16 patients (18%) who felt globally improved (“much better” or “very much better”). Therefore, we conclude that our placebo treatment, NaCl injections, are not an adequate treatment either.

In our view, the collective findings of this RCT suggest that addressing CPP may require a more comprehensive, multidisciplinary approach. Such an approach should consider various contributing factors beyond the physical aspect, including psychological and social factors.

### 4.1 Strengths

This study possesses several strengths. Firstly, it utilises a randomised design and employs blinding techniques. Additionally, the study includes a notable 26‐week follow‐up duration, enabling assessment of effects after recovery from the inhibition of acetylcholine release by BTA. Moreover, participants had previously undergone first‐line PFMT, and the additional PFMT was aimed at breaking the vicious cycle of pain and muscle contraction. Furthermore, the study benefits from increased generalisability through nationwide participation from secondary and tertiary care hospitals.

### 4.2 Limitations

There are several limitations to this study. Firstly, participants inclusion began 2 months prior to the global COVID‐19 pandemic reaching the Netherlands. Strict regulations extended the inclusion period by approximately 8 months and led to the cancellation of 19 in‐person scheduled follow‐up study visits. We have no indication, however, that this has influenced study results. Secondly, although the calculated sample size was achieved, the power calculation assumed no response to placebo injections. However, a combination of the possible placebo effect, wet‐ and/or dry‐needling effect and PFMT might have contributed to pain relief in the placebo group with saline injection. Thirdly, the painDETECT questionnaire is commonly used for identifying neuropathic pain and is not specific to CPP. Additionally, the questionnaire is not validated for the separate analysis of individual questions as we have performed. Fourthly, envelope randomisation was employed, potentially introducing bias. However, stringent protocols were followed and baseline characteristics were similar between groups, Finally, this study represents the Dutch patient population. While the generalisability of outcomes to populations with different characteristics remains uncertain, comparison with other studies do not indicate international divergence [14, 27].

### 4.3 Future Research

An open‐label extension study was conducted for participants who had received placebo, and not meeting the primary endpoint of this RCT. Moreover, results of a 1‐year follow‐up will follow. Future studies could focus on dyspareunia.

## 5 Conclusion

Based on the absence of a difference between groups in average pain score and the patients' global impression of improvement, this study does not support BTA injections in the management of CPP in women. Further research is needed to better define the patient subgroups most likely to respond to this treatment and to ensure that its use is appropriately targeted in clinical practice.

## Author Contributions

**Melle A. Spruijt:** study design, Data Analysis and Interpretation, manuscript drafting. **Wenche M. Klerkx:** intellectual contribution and expertise, concept and study design, Data Acquisition, Editing and Revising manuscript. **Kim Notten:** intellectual contribution and expertise, Data Acquisition. **Hugo van Eijndhoven:** intellectual contribution and expertise, Data Acquisition, Editing and Revising manuscript. **Leonie Speksnijder:** intellectual contribution and expertise, Data Acquisition, Editing and Revising manuscript. **Manon H. Kerkhof:** intellectual contribution and expertise, Data Acquisition, Editing and Revising manuscript. **Kirsten B. Kluivers:** intellectual contribution and expertise, Editing and Revising manuscript.

## Conflicts of Interest

The Botulinum toxin A was provided by Allergan/Abbvie (unrestricted).

## Supporting information


Table S1.

Table S1.

## Data Availability

The data that support the findings of this study are available on request from the corresponding author. The data are not publicly available due to privacy or ethical restrictions.
