# Supplementary material for: The Efficacy of Botulinum Toxin A Injection in Pelvic Floor Muscles in Chronic Pelvic Pain Patients: A Double‐Blinded Randomised Controlled Trial
Source: BJOG. 2024 Nov 13;132(3):297–305. doi: 10.1111/1471-0528.17991 (PMC11704059; doi:10.1111/1471-0528.17991)
Supplement: Supplementary file 1 — Table S1. [file BJO-132-297-s002.docx]

**Online only:**

| **Table S1. Secondary outcomes** | | | |
| --- | --- | --- | --- |
| Outcome | **Baseline**  BTA n=47; Placebo n=47 | **26** **weeks**  BTA n=46; Placebo n=44 | p-value |
| **PainDETECT total score ^a^** |  |  |  |
| BTA  Placebo  BTA vs. placebo | 14.0 [12.2 – 15.9]  14.5 [12.7 – 16.3] | 13.3 [11.5 – 15.1]  13.8 [11.9 – 15.6] | 0.89 |
| **VAS current score ^b^** |  |  |  |
| BTA  Placebo  BTA vs. placebo | 5.0 [4.2 – 5.7]  6.1 [5.4 – 6.9] | 4.8 [4.1 – 5.6]  5.0 [4.2 – 5.8] | 0.22 |
| **VAS score, maximum ^b^** |  |  |  |
| BTA  Placebo  BTA vs. placebo | 8.6 [8.2 – 9.1]  8.6 [8.1 – 9.0] | 8.1 [7.6 – 8.5]  7.8 [7.4 – 8.3] | 0.42 |
| **VAS score, minimum ^b^** |  |  |  |
| BTA  Placebo  BTA vs. placebo | 3.3 [2.7 – 4.0]  3.6 [2.9 – 4.2] | 3.0 [2.3 – 3.6]  3.1 [2.4 – 3.7] | 0.21 |
| **EQ health scale ^c^** |  |  |  |
| BTA  Placebo  BTA vs. placebo | 54.5 [48.6 – 60.4]  50.1 [44.2 – 56.0] | 58.9 [52.9 – 64.9]  59.0 [53.0 – 65.0] | 0.24 |
| **HADS depression scale^d^** |  |  |  |
| BTA  Placebo  BTA vs. placebo | 6.5 [5.3 – 7.7]  7.6 [6.4 – 8.9] | 6.1 [4.9 – 7.3]  6.7 [5.5 – 7.9] | 0.09 |
| **HADS anxiety scale ^d^** |  |  |  |
| BTA  Placebo  BTA vs. placebo | 8.0 [6.9 – 9.1]  7.8 [6.7 – 8.9] | 6.4 [5.3 – 7.5]  7.1 [6.0 – 8.2] | 0.26 |
| **Pain catastrophizing scale (PCS)^e^** |  |  |  |
| BTA  Placebo  *BTA vs. placebo | 23.4 [20.0 – 26.9]  24.0 [20.5 – 27.5] | 18.1 [14.6 – 21.6]  18.3 [14.8 – 21.9] | 0.45 |
| **PFIQ-7 total score ^f^** |  |  |  |
| BTA  Placebo  Botox vs. placebo | 102.7 [83.4 – 121.9]  118.9 [99.4 – 138.4] | 88.8 [69.4 – 108.3]  96.0 [76.3 – 115.7] | 0.85 |
| **PFDI-20 total score ^g^** |  |  |  |
| BTA  Placebo  BTA vs. placebo | 91.9 [78.1 – 105.6]  97.5 [83.8 – 111.3] | 83.5 [69.7 – 97.2]  85.9 [72.1 – 99.8] | 0.55 |
| Data presented as mean (95% confidence interval) or p-value as measured with linear mixed models and ANOVA.  ^a^ A lower score indicates nociceptive pain (<12) and a higher score indicates neuropathic pain (>19). In between (13-18) there is possible neuropathic pain.  ^b^ Visual analog scale current pain score (0-10) at time of questionnaire.  ^c^ EQ health scale: records the patients self-rated health on a vertical visual analog scale (0-100), higher score indicates a better self-rated health  ^d^ Hospital anxiety and depression scale, categorised in normal (0-7), borderline normal (8-10) and abnormal (11-21)  ^e^ Pain catastrophizing scale, a higher score indicates a greater degrees of pain catastrophizing. A total score of >30 represents a clinically significant level of pain catastrophizing.  ^f^ Pelvic floor impact questionnaire, higher score indicate more impact on daily activity  ^g^ Pelvic floor distress inventory (scale 0-300), higher score indicate more symptom distress  ^h^ Pelvic Organ Prolapse/Incontinence Sexual Questionnaire IUGA-Revised, a higher score indicates a better sexual function  ^I^ Measured by MAPLe (μV) | | | |
