# Supplementary material for: The Efficacy of Botulinum Toxin A Injection in Pelvic Floor Muscles in Chronic Pelvic Pain Patients: A Double‐Blinded Randomised Controlled Trial
Source: BJOG. 2024 Nov 13;132(3):297–305. doi: 10.1111/1471-0528.17991 (PMC11704059; doi:10.1111/1471-0528.17991)
Supplement: Supplementary file 2 — Table S1. [file BJO-132-297-s001.docx]

*Statistical Analysis Plan*

**Botox study**

**The efficacy of botulinum toxin A injection in pelvic floor muscles in chronic pelvic pain patients: a double-blinded randomized controlled trial**

| **EUDRA CT no.**  **Dutch Clinical trial registry no.** | 2017-001296-23 |
| --- | --- |
| **Principal investigator, centre** | Dr. Wenche M. Klerkx, st Antonius  Dr. Kirsten B. Kluivers, Radboud UMC |
| **Coordinating investigator** | Drs. Melle A. Spruijt |
| **Sponsor** | Radboudumc |
| **SAP version, date** | Version 5, march 2024 |
| **Trial methodologist** | Hans Kelder |
| **SAP author** | Melle Spruijt |

Inhoudsopgave

[**1.** **List of abbreviations** 3](#_Toc141888108)

[**2.** **Introduction** 4](#_Toc141888109)

[a. Background 4](#_Toc141888110)

[b. Objective 4](#_Toc141888111)

[**3.** **Endpoints** 4](#_Toc141888112)

[a. Primary endpoints 4](#_Toc141888113)

[b. Secondary endpoints 4](#_Toc141888114)

[**4.** **Study methods** 5](#_Toc141888115)

[a. Study design 5](#_Toc141888116)

[b. Study population 5](#_Toc141888117)

[c. Inclusion criteria 5](#_Toc141888118)

[d. Exclusion criteria 5](#_Toc141888119)

[e. Treatment of subjects 6](#_Toc141888120)

[f. Study procedures 6](#_Toc141888121)

[g. Randomization procedure and blinding 6](#_Toc141888122)

[h. Replacement of individual subjects after withdrawal 7](#_Toc141888123)

[**5.** **Sample-size** 7](#_Toc141888124)

[**6.** **Analysis considerations** 7](#_Toc141888125)

[a. Intention-to-treat 7](#_Toc141888126)

[b. Covariates and Subgroups 7](#_Toc141888127)

[c. Missing data 8](#_Toc141888128)

[d. Interim Analyses and Data Monitoring 8](#_Toc141888129)

[**7.** **Efficacy analyses** 8](#_Toc141888130)

[a. Timing of final statistical analysis 8](#_Toc141888131)

[b. Primary and secondary outcome analysis 8](#_Toc141888132)

[**8.** **Safety analyses** 9](#_Toc141888133)

[a. Adverse events 9](#_Toc141888134)

[b. Deaths, Serious Adverse Events and other significant Adverse events 9](#_Toc141888135)

[**9.** **Other analyses** 9](#_Toc141888136)

[**10.** **Comparison to study protocol** 9](#_Toc141888137)

[**11.** **Presentation of study results** 9](#_Toc141888138)

[a. Recruitment 9](#_Toc141888139)

[b. Protocol violations 9](#_Toc141888140)

[c. Baseline characteristics 9](#_Toc141888141)

[d. Primary outcome 10](#_Toc141888142)

[e. Secondary outcomes 10](#_Toc141888143)

[**12.** **Definition of variables** 10](#_Toc141888144)

[a. Primary outcome 10](#_Toc141888145)

[b. Secondary outcomes 10](#_Toc141888146)

[**13.** **Tables** 11](#_Toc141888147)

[a. Baseline characteristics 11](#_Toc141888148)

[b. Primary outcome 12](#_Toc141888149)

[c. Safety outcomes 12](#_Toc141888150)

[d. Secondary outcomes 13](#_Toc141888151)

[**14.** **Figures** 14](#_Toc141888152)

[a. Flowchart of participants 14](#_Toc141888153)

[**15.** **References** 14](#_Toc141888154)

1. **List of abbreviations**

- BTA Botulinum toxin A
- CPP Chronic pelvic pain
- EQ-5D Quality of life questionnaire
- HADS Hospital Anxiety and Depression Scale
- MAPle Multiple Array Probe Leiden
- PCS Pain Catastrophizing Scale
- PFDI-20 Pelvic Floor Distress Inventory 20
- PFIQ-7 Pelvic Floor Impact Questionnaire 7
- PGI-I Patient Global Impression of Improvement
- PISQ-IR Pelvic Organ Prolapse/Incontinence Sexual Questionnaire, IUGA-Revised
- RCT Randomized controlled trial
- QoL Quality of Life
- VAS Visual Analogue Scale

1. **Introduction**
   1. Background

Chronic pelvic pain is common, affecting 15% of women aged 18-50^1^. Pelvic floor muscle spasms resulting in chronic pelvic pain may occur as a primary event or secondary to a physical, psychological or pathological factor. First-line treatment consists of pelvic floor physiotherapy. When first-line treatment fails, more invasive interventions can be done. One previously published intervention is injection with botulinum toxin A (BTA) in the pelvic floor muscles. It produces a localized, partial, and reversible chemical denervation of the muscle which results in localized muscle weakness or paralysis. There is some evidence that injection of BTA in the hypertonic pelvic floor muscles decreases pelvic pain in patients with therapy resistant chronic pelvic pain; however this is not investigated in a randomized controlled trial.

- 1. Objective

The primary objective of this randomized, double-blind, placebo controlled trial was to compare the effect of BTA injections vs. placebo injections on pelvic pain in women with chronic pelvic pain.

1. **Endpoints**
   1. Primary endpoints

Decrease of chronic pelvic pain, measured by a decrease in visual analog scale score (VAS score 0-10) with 33% and a patient global impression of improvement score (PGI-I) of 1 or 2 (better or much better) at 26 weeks after injection.

- 1. Secondary endpoints
     1. Subjective outcomes (patient reported outcomes)
- pelvic floor distress inventory (PFDI-20)^2^
- pelvic floor impact questionnaire (PFIQ-7)^2^
- quality of life (EQ-5D)^3^
- painDETECT^4^
- pain catastrophizing scale (PCS)^5^
- hospital anxiety and depression scale (HADS)^6^
- sexual function (PISQ-IR)^7^
- Visual analog scale (VAS) score ^8^
  - 1. Anatomical outcomes
- Pelvic floor hypertonicity measured by the MAPLe device
  - 1. Other outcomes
- A cost-effectiveness analysis will be performed in case of a significant improved VAS score and quality of life.
  1. One year follow-up

To evaluate the safety and efficacy of a BTA injection one year after BTA injection.

- 1. Open label extension study

To evaluate the safety of BTA injection 26 weeks after the placebo injection in participants who did not reach the primary endpoint.

1. **Study methods**
   1. Study design

Double-blinded randomized placebo-controlled trial.

- 1. Study population

Patients over 16 years with >6 months of chronic pelvic pain with pelvic floor muscle hypertonicity refractory to first-line pelvic floor physiotherapy, and in whom no anatomical cause was found.

* Definition by the International Continence Society: Chronic pelvic pain is characterized by persistent pain lasting longer than 6 months or recurrent episodes of abdominal/pelvic pain, hypersensitivity or discomfort often associated with elimination changes, and sexual dysfunction often in the absence of organic etiology^9^.

- 1. Inclusion criteria
- Female, >16 years
- Chronic pelvic pain according to the ICS with or without dyspareunia
- Vaginal examination with one finger possible
- Pelvic floor hypertonicity measured by physical examination by registered pelvic floor physiotherapist and MAPLe
- Previous physical therapy with registered physical therapist was unsuccessful
- Good understanding of Dutch language
- Willing to provide informed consent
  1. Exclusion criteria
- (wish for) Pregnancy/lactation during study period
- Previous pelvic floor BTA treatment
- Known hypersensitivity to BTA
- History of neuromuscular or bleeding disorders
  1. Treatment of subjects

The pelvic floor muscles will be injected with either 100 IU BTA or placebo.

- 1. Study procedures

The subjects will undergo the following procedures after signing informed consent:

- Physical examination by a gynecologist and a physical therapist
- MAPLe measurement by a physical therapist
- The patient preference questionnaire will be filled in
- The validated questionnaires will be filled in at baseline, 4, 8, 12 and 26 weeks after injection
- Injection of study medication or placebo in pelvic floor muscles
- Standardized physical therapy by an experienced registered pelvic floor physical therapist during the study period
- Deblinding of subjects who did not meet the primary endpoint and offer them a second injection with BTA.
- As part of the Open-label Extension Study, subject will fill in validated questionnaires at 4, 8, 12 and 26 weeks after BTA injection.
- One year follow-up questionnaires will be filled in after the primary BTA/placebo injection or one year after the BTA injection in case of the Open-label Extension Study.
  1. Study medication and preparation

The investigational medicinal product (IMP) will either be Botulinum Toxin Type A 100IU or 0.9% NaCl (placebo).

The BTA and placebo will be supplied from site stock, where it is stored according to Good Distribution Practice (GDP ), recommended respectively in Dutch hospital pharmacies and outpatient clinics. The drugs will become investigational medicinal products only when they are made ready for administration.

The independent health professional, not involved in the study or treatment of the patient, will prepare the syringe with the investigational medicinal product under aseptic conditions just before administration. The independent health professional will dissolve 100IU BTA in 6cc 0.9% NaCl in a syringe or only 6cc 0.9% NaCl in a syringe, depending on the treatment arm. This process will always be verified by a second, independent person (controller) to assure that the process is done correctly.

- 1. Randomization procedure and blinding

Participants were randomly allocated to BTA injection or placebo injection in a 1:1 ratio. The 94 sealed opaque envelopes were randomly divided across the inclusion centres and opened on the day of randomization. The gynaecologist, physical therapist and participant were blinded to the treatment arm. Participants who did not meet the primary endpoint (33% reduction VAS score or PGI-I 1 or 2) were deblinded after 26 weeks. Participants who received placebo injection were offered to participate in our open label extension study and receive BTA injection concurrent with pelvic floor physical therapy.


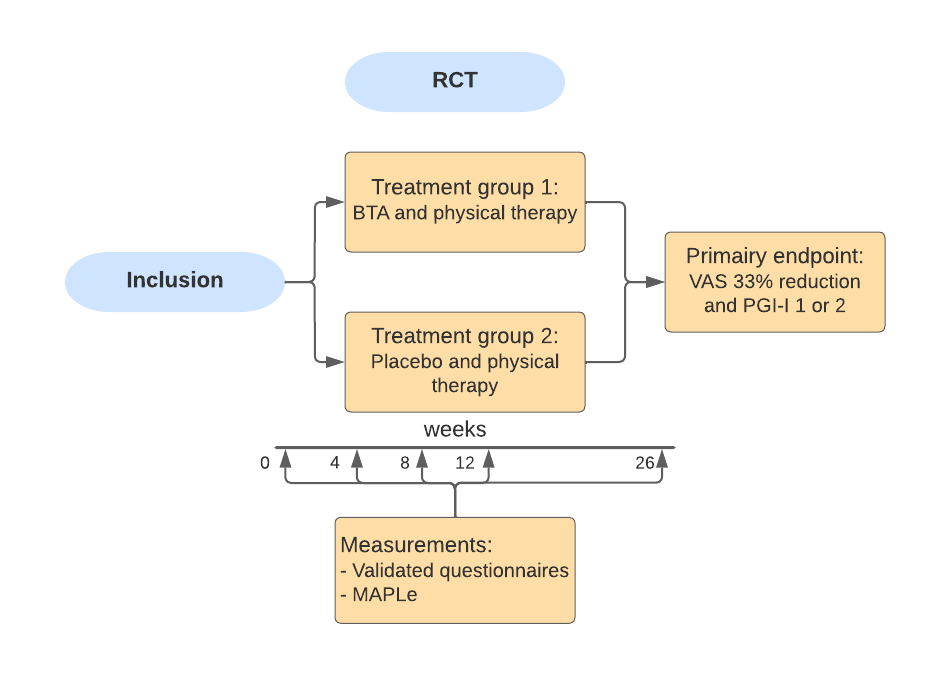


Figure 1. flow-chart RCT


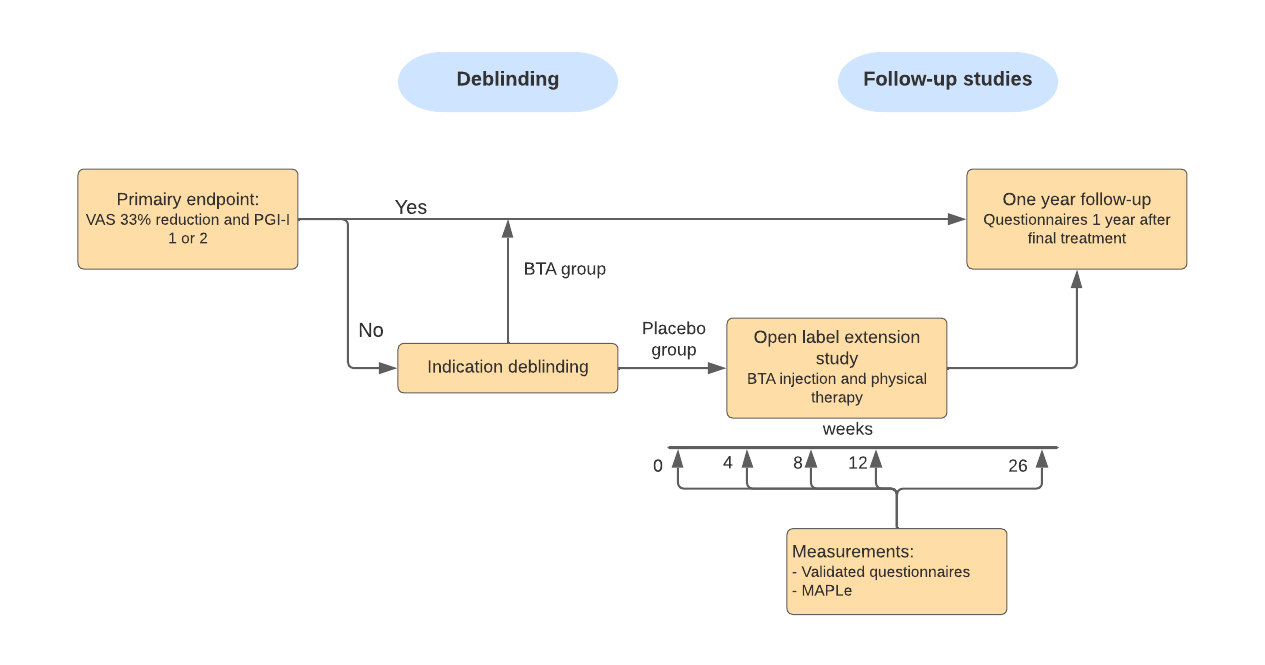


Figure 2. flow-chart follow-up studies after ending of RCT: one year follow-up and open-label extension study

- 1. Replacement of individual subjects after withdrawal

If withdrawal occurs before the injection, these patients will be replaced in order to reach the desired statistical power. If withdrawal occurs during the follow-up period, between injection and before the endpoint of 26 weeks, the data will be censored at the last available time-point. Analyses of available data will be performed in the intention to treat group.

1. **Sample-size**

The ANCOVA procedure^10^ showed that to detect a statistical significant 33% difference in pain scores between the intervention group and the control group, a sample size of 94 individuals was required for this trial (47 per group), with 80% power and assuming a two-sided alpha of 0.05.

1. **Analysis considerations**

To evaluate the effect of BTA on the VAS score during follow-up all statistical analysis will be performed according to intention to treat principle.

- 1. Analysis of groups

The intention-to-treat (ITT) population is the as-randomized population. This will consist of all patients who have given consent and have been allocated to one of the two treatments, irrespective of treatment received.

- 1. Covariates and Subgroups

Subgroup analyses are planned to investigate the possible effect on the outcome of treatment of the following possible pre-defined interaction terms:

- Menopausal status (yes vs. no)
- Negative sexual experience (yes vs. no)
- Depression scale at baseline (HADS) (scale 0-21 points)
- Previous operations (Abdominal/pelvic operation vs other)
- Parity (absolute numbers)
- Use of pain opioids (yes vs. no)
- Sexual activity (yes vs. no)

Subgroup analyses will be conducted by adding an interaction term to the model and testing for the statistical significance of this interaction term. We will subsequently examine the likelihood that we have missed any relevant interaction terms through a likelihood ratio test for all baseline characteristics available.

In version 5 of our statistical analysis plan we added one subgroup to our analysis:

- Duration of CPP (<1 year vs. >1 year)

Furthermore, due to our study design (double-blind randomized controlled trial), we determined that covariates did not add value to our analysis.

- 1. Missing data

We will use mixed models for repeated measurements and its ability to handle missing data effectively. Before analyzing our results using mixed models for repeated measurements, the following questions will be answered:

1. Is the occurrence of missing data random, or is there a discernible pattern among these individuals?
2. What is the source of the missing data?
3. Is there any correlation with specific variables?
4. Can we identify a pattern in the missing data?

Based on the answers to these questions, the potential options for the pattern of missing data are as followed: Missing completely at random (MCAR), missing not at random (MNAR), and missing at random (MAR).

In case of additional analyses not performed using mixed models, missing data will be examined and if necessary randomly imputated.

Patients who are lost to follow-up will be censored at the last visit on which all assessments were completed.

- 1. Interim Analyses and Data Monitoring

No interim analyses are planned.

1. **Efficacy analyses**
   1. Timing of final statistical analysis

The statistical analyses of the primary outcome and secondary outcomes (measured 26 weeks after treatment) will be performed after a minimum of 26 weeks have elapsed from the inclusion of the final patient in the study and after data cleaning for these outcomes has been completed.

The statistical analysis of the follow-up study will be performed 26 weeks after inclusion of the final patient and data cleaning for these outcomes has been completed.

- 1. Primary and secondary outcome analysis

The null hypothesis entails that there is no significant difference in decrease of chronic pelvic pain scores 26 weeks after treatment between the intervention group receiving BTA injections and the control group receiving placebo injections among women with chronic pelvic pain.

VAS score Analysis:

- Mixed models for repeated measurements will be used. VAS scores will be compared over time between groups
- Primary endpoint: Percentage difference in VAS score between baseline and 26 weeks post-treatment will be calculated for each group.

PGI-I Analysis:

- Mixed models for repeated measurements will be used.
- PGI-I scores will be analyzed to identify differences between groups over time.
- Primary endpoint: the primary outcome of PGI-I scores being 1 or 2 (presented as median and interquartile (IQ) range) will be compared between groups.

Secondary outcome analysis

Questionnaire Scores:

- The total scores of all questionnaires will be analyzed over time using mixed models for repeated measurements.
- Analysis will involve assessing changes over time between groups MAPLe
- EMG signals will be analysed using mixed models for repeated measurements

The analyses will be performed with IBM SPSS Statistics (version 29, Armonk, New York, United States) and R.

In our version 5 statistical analysis, we found that adjusting for baseline and age did not enhance our analysis, given our study's design as a double-blind randomized controlled trial. Therefore, we removed this adjustment from our statistical analysis plan.

1. **Safety analyses**
   1. Adverse events

Adverse event data will be analysed as allocated (intention-to-treat) and 95% confidence interval will be calculated where possible. We do however not foresee a high number of adverse events and in that case adverse events will be mentioned in a table or the text.

- 1. Deaths, Serious Adverse Events and other significant Adverse events

Death/serious adverse events will be described by treatment group.

1. **Other analyses**

Subgroup analysis mentioned in section 6 is seen as hypothesis generating.

1. **Comparison to study protocol**

The current analysis plan is largely based on the published study protocol^11^. Slight changes were made and these are listed below:

- Not only IBM SPSS Statistics for Windows, Version 26.0. will be used, but also R.
- We will not use Kaplan Meier to estimate the success rates at 26 weeks of follow-up. Instead we will use the mixed models for repeated measurements.

1. **Presentation of study results**
   1. Recruitment

The flow of recruitment of study participants will be presented in the CONSORT flow diagram^12^.

- 1. Protocol violations

All protocol violations will be line-listed by treatment group.

- 1. Baseline characteristics

Baseline characteristics of the total randomized (intention-to-treat) population will be presented following the format of the mock table provided in section 13. Discrete outcomes will be presented using absolute numbers and percentages, while continuous outcomes will be presented as means with standard deviations or medians with interquartile ranges.

- 1. Primary outcome

The primary outcome will be presented for the total population as randomised (intention-to-treat). Data will be presented using absolute numbers with percentages for discrete outcomes. Relative Risk or mean difference will be presented together with 95% confidence interval, and p-value otherwise as appropriate.

- 1. Secondary outcomes

The secondary outcomes will be presented for the total population as randomised (intention-to-treat). Data will be presented using absolute numbers with percentages for discrete outcomes. Relative Risk or mean difference will be presented together with 95% confidence interval, and p-value otherwise as appropriate.

1. **Definition of variables**
   1. Primary outcome

- VAS sore: we will use the painDETECT questionnaire to determine patients VAS score at every time point. We will use the following question out of the painDETECT questionnaire:
  - - - How strong was the pain during the past 4 weeks on average?

We believe that the average pain score over the past 4 weeks provides a more accurate reflection of the current situation than the pain score at the specific moment itself. The latter is influenced by various other factors.

- PGI-I: we will measure our patients interpretation of symptom changes following treatment. A score of 1 or 2 will be considered clinically significant and relevant.
  1. Secondary outcomes
     1. Patient reported outcomes based on questionnaires. Scores of the questionnaires will be calculated in line with the questionnaire instruction.
- pelvic floor distress inventory (PFDI-20): raw outcomes will be transformed into the following results: Pelvic Organ Prolapse Distress Inventory-6 (POPDI-6), Colorectal-Anal distress Inventory 8 (CRAB-8), Urinary distress Inventory 6 (UDI-6) and the PFDI total score
- pelvic floor impact questionnaire (PFIQ-7): raw outcomes will be transformed into the following results: Urinary impact questionnaire (UIQ-7), Colorectal-anal impact questionnaire CRAIQ-7, Pelvic organ prolaps impact questionnaire (POPIQ-7) and the PFQI-7 total score.
- quality of life (EQ-5D)
- painDETECT: raw outcomes will be transformed into a painDETECT total score and will be categorized into negative, unclear and positive.
- pain catastrophizing scale (PCS): raw outcomes will be transformed into a pain catastrophizing total score
- hospital anxiety and depression scale (HADS): raw outcomes will be transformed into: depression score and anxiety score
- the Pelvic Organ Prolapse/Incontinence Sexual Questionnaire, IUGA-Revised (PISQ-IR): raw outcomes will be transformed into the following results:
  - 1. Multiple Array Probe Leiden (MAPLe): this method involves measuring the EMG signals closest to the pelvic floor, allowing us to assess pelvic floor muscle tone at 24 different locations. EMG signals will be recorded during periods of rest, endurance and the maximum voluntary contraction. Measurements are standardized through a protocol. We will specifically focus on the EMG measurements taken during rest. This particular measurement is considered the most reliable, as opposed to the other measurements which are derived as averages of three to ten individual measurements.

1. **Tables**
   1. Baseline characteristics

| **Table 2.** Baseline characteristics by treatment group | | |
| --- | --- | --- |
| **Characteristics** | BTA  (n=x) | Placebo  (n=x) |
| Age, median [IQR], y | Median [IQR] | Median [IQR] |
| Ethnicity: Caucasian (%) | NNN% | NNN% |
| Body mass index (BMI)^a^ , median [IQR] | Median [IQR] | Median [IQR] |
| Parity, No. (%)  - Nulliparous  - Primiparous  - Multiparous | NNN%  NNN%  NNN% | NNN%  NNN%  NNN% |
| Menopausal, No. (%) | NNN% | NNN% |
| Relationship status: in a relationship, No. (%) | NNN%^Ω^ | NNN% |
| Sexually active, No. (%) | NNN% | NNN% |
| History of sexual abuse, No. (%) | NNN% | NNN% |
| Smoking, No. (%) | NNN% | NNN% |
| Surgical history ^b^, No. (%)  - Abdominal surgery  - Laparoscopic surgery  - Prolapse surgery  - Other | NNN%  NNN%  NNN%  NNN% | NNN%  NNN%  NNN%  NNN% |
| Pain score at baseline^c^, mean [SD] | Median [IQR] | Median [IQR] |
| Use of pain medication, No. (%)  - No use of pain medication/ paracetamol  - Non-steroidal anti-inflammatory drugs (NSAIDs)  - Neuropathic pain medication^d^  - Opioids | NNN%  NNN%  NNN%  NNN% | NNN%  NNN%  NNN%  NNN% |
| Other chronic pain syndromes (not CPP)^b,e^, No. (%) | NNN% | NNN% |
| ^a^ Calculated as weight in kilograms divided by height in meters squared  ^b^ Data were collected at the study inclusion visit and were supplemented by study research nurses through medical record review.  ^c^ Average pain score past four weeks, scale 0-10  ^d^ Amitriptyline, gabapentin, pregabalin and nortriptyline.  ^e^ Including: fibromyalgia, piriformis syndrome, irritated bowel syndrome, interstitial cystitis, provoked vulvodynia | | |

- 1. Primary outcome

**Table 3. Primary outcomes.**

| Outcomes | BTA | Placebo | p-value |
| --- | --- | --- | --- |
| **Pain score** | N=xx | N=xx |  |
| Baseline | Mean (95% CI) | Mean (95% CI) |  |
| 4 weeks follow-up  8 weeks follow-up  12 weeks follow-up  26 weeks follow-up  BTA vs placebo*  Primary endpoint: 33% reduction in pain score, No. (%) | Mean (95% CI) Mean (95% CI) Mean (95% CI) Mean (95% CI)  NNN% | Mean (95% CI)  Mean (95% CI)  Mean (95% CI)  Mean (95% CI)  NNN% | X  X |
| **PGI-I** | N=xx | N=xx |  |
| 4 weeks follow-up  8 weeks follow-up  12 weeks follow-up  26 weeks follow-up  BTA vs placebo*  Primary endpoint: PGI-I 1 or 2, No. (%) | Mean (95% CI)  Mean (95% CI)  Mean (95% CI)  Mean (95% CI)  NNN% | Mean (95% CI)  Mean (95% CI)  Mean (95% CI)  Mean (95% CI)  NNN% | X  X |
| Data are presented as mean [95% CI] unless stated otherwise  *Pain score: average pain score last 4 weeks, scale 0-10.  **Based on linear mixed models including all time points except for baseline. Controlling for pain score (average past 4 weeks) at baseline and age at baseline.  *** p value for interaction | | | |

- 1. Secondary outcomes

| Outcome | Baseline n=xx | 26 weeks  n=xx | p-value |
| --- | --- | --- | --- |
| **PainDETECT total score** ^a^ |  |  |  |
| BTA  Placebo  *Botox vs. placebo | Mean (95% CI)  Mean (95% CI) | Mean (95% CI)  Mean (95% CI) | X |
| **Current VAS score^b^** |  |  |  |
| BTA  Placebo  *Botox vs. placebo | Mean (95% CI)  Mean (95% CI) | Mean (95% CI)  Mean (95% CI) |  |
| **EQ5D VAS score^c^** |  |  |  |
| BTA  Placebo  *Botox vs. placebo | Mean (95% CI)  Mean (95% CI) | Mean (95% CI)  Mean (95% CI) | X |
| **HADS depression scale^d^** |  |  |  |
| BTA  Placebo  *Botox vs. placebo | Mean (95% CI)  Mean (95% CI) | Mean (95% CI)  Mean (95% CI) | X |
| **HADS anxiety scale^d^** |  |  |  |
| BTA  Placebo  *Botox vs. placebo | Mean (95% CI)  Mean (95% CI) | Mean (95% CI)  Mean (95% CI) | X |
| **Pain catastrophizing scale (PCS)^e^** |  |  |  |
| BTA  Placebo  *Botox vs. placebo | Mean (95% CI)  Mean (95% CI) | Mean (95% CI)  Mean (95% CI) | X |
| **PFIQ-7 total score^f^** |  |  |  |
| BTA  Placebo  *Botox vs. placebo | Mean (95% CI)  Mean (95% CI) | Mean (95% CI)  Mean (95% CI) | X |
| **PFDI-20 total score^g^** |  |  |  |
| BTA  Placebo  *Botox vs. placebo | Mean (95% CI)  Mean (95% CI) | Mean (95% CI)  Mean (95% CI) | X |
| **PISQ-IR total score^h^** |  |  |  |
| BTA  Placebo  *Botox vs. placebo | Mean (95% CI)  Mean (95% CI) | Mean (95% CI)  Mean (95% CI) | X |
| **Pelvic Floor Resting Tone^i^** |  |  |  |
| BTA  Placebo  *Botox vs. placebo | Mean (95% CI)  Mean (95% CI) | Mean (95% CI)  Mean (95% CI) | X |

Data presented as mean (95% confidence interval) or p-value.

* Based on linear mixed models. Controlling for average pain and age at baseline.

^a^ A lower score indicates nociceptive pain (<12) and a higher score indicates neuropathic pain (>19). In between (13-18) there is possible neuropathic pain.

^b^ Visual analog scale score (0-10) at time of questionnaire.

^c^ EQ VAS component: records the patients self-rated health on a vertical visual analog scale (0-100), higher score indicates a better self-rated health

^d^ Hospital anxiety and depression scale, categorized in normal (0-7), borderline normal (8-10) and abnormal (11-21)

^e^ Pain catastrophizing scale, a higher score indicates a greater degrees of pain catastrophizing. A total score of >30 represents a clinically significant level of pain catastrophizing.

^f^ Pelvic floor impact questionnaire, higher score indicate more impact on daily activity

^g^ Pelvic floor distress inventory (scale 0-300), higher score indicate more symptom distress

^h^ Pelvic Organ Prolapse/Incontinence Sexual Questionnaire, IUGA-Revised, a higher score indicates a better sexual function

^I^ Measured by MAPLe

1. **Figures**
   1. Flowchart of participants


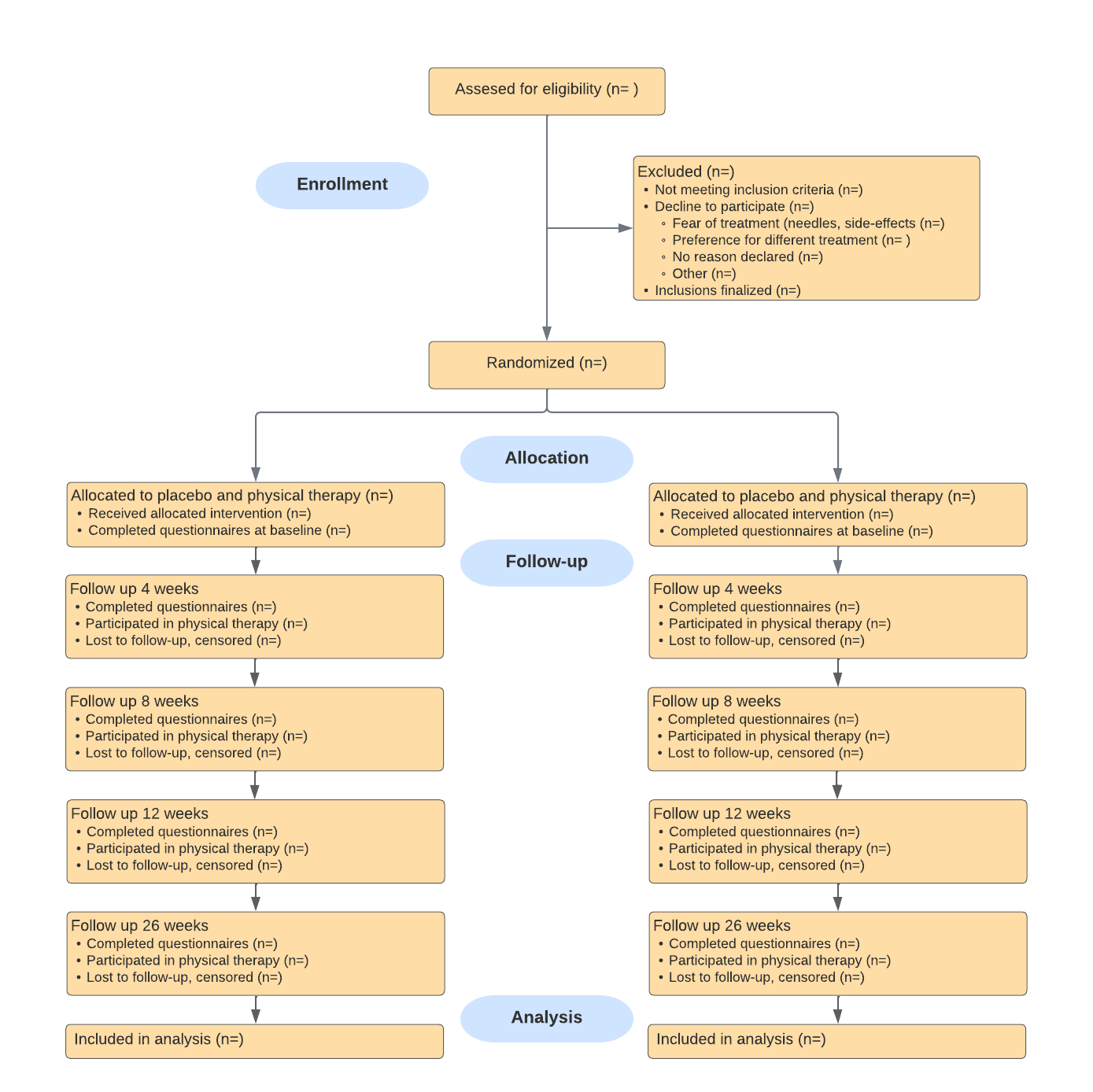


1. **References**
2. Mathias SD, Kuppermann M, Liberman RF, Lipschutz RC, Steege JF. Chronic pelvic pain: prevalence, health-related quality of life, and economic correlates. Obstet Gynecol. 1996 Mar;87(3):321-7. doi: 10.1016/0029-7844(95)00458-0. PMID: 8598948.
3. Barber MD, Walters MD, Bump RC. Short forms of two condition-specific quality-of-life questionnaires for women with pelvic floor disorders (PFDI-20 and PFIQ-7). Am J Obstet Gynecol. 2005 Jul;193(1):103-13. doi: 10.1016/j.ajog.2004.12.025. PMID: 16021067.
4. Rabin R, de Charro F. EQ-5D: a measure of health status from the EuroQol Group. Ann Med. 2001 Jul;33(5):337-43. doi: 10.3109/07853890109002087. PMID: 11491192.
5. Freynhagen R, Tölle TR, Gockel U, Baron R. The painDETECT project - far more than a screening tool on neuropathic pain. Curr Med Res Opin. 2016 Jun;32(6):1033-57. doi: 10.1185/03007995.2016.1157460. Epub 2016 Mar 11. PMID: 26907456.
6. Darnall BD, Sturgeon JA, Cook KF, Taub CJ, Roy A, Burns JW, Sullivan M, Mackey SC. Development and Validation of a Daily Pain Catastrophizing Scale. J Pain. 2017 Sep;18(9):1139-1149. doi: 10.1016/j.jpain.2017.05.003. Epub 2017 May 19. PMID: 28528981; PMCID: PMC5581222.
7. Bjelland I, Dahl AA, Haug TT, Neckelmann D. The validity of the Hospital Anxiety and Depression Scale. An updated literature review. J Psychosom Res. 2002 Feb;52(2):69-77. doi: 10.1016/s0022-3999(01)00296-3. PMID: 11832252.
8. van Dongen H, van der Vaart H, Kluivers KB, Elzevier H, Roovers JP, Milani AL. Dutch translation and validation of the pelvic organ prolapse/incontinence sexual questionnaire-IUGA revised (PISQ-IR). Int Urogynecol J. 2019 Jan;30(1):107-114. doi: 10.1007/s00192-018-3718-z. Epub 2018 Jul 14. PMID: 30008079.
9. Jensen MP, Chen C, Brugger AM. Interpretation of visual analog scale ratings and change scores: a reanalysis of two clinical trials of postoperative pain. J Pain. 2003 Sep;4(7):407-14. doi: 10.1016/s1526-5900(03)00716-8. PMID: 14622683.
10. Doggweiler R, Whitmore KE, Meijlink JM, Drake MJ, Frawley H, Nordling J, et al. A standard for terminology in chronic pelvic pain syndromes: a report from the chronic pelvic pain working group of the international continence society. Neurourol Urodyn. 2017;36(4):984–1008. https:// doi. org/ 10. 1002/ nau. 23072.
11. Borm GF, Fransen J, Lemmens WAJG. A simple sample size formula for analysis of covariance in randomized clinical trials. Journal of Clinical Epidemiology. 2007:60: 1234-1238.
12. Spruijt M, Kerkhof M, Rombouts M, Brohet R, Klerkx W. Efficacy of botulinum toxin A injection in pelvic floor muscles in chronic pelvic pain patients: a study protocol for a multicentre randomised controlled trial. BMJ Open. 2023 Jul 7;13(7):e070705. doi: 10.1136/bmjopen-2022-070705. PMID: 37419648; PMCID: PMC10335469.
13. Butcher NJ, Monsour A, Mew EJ, Chan AW, Moher D, Mayo-Wilson E, Terwee CB, Chee-A-Tow A, Baba A, Gavin F, Grimshaw JM, Kelly LE, Saeed L, Thabane L, Askie L, Smith M, Farid-Kapadia M, Williamson PR, Szatmari P, Tugwell P, Golub RM, Monga S, Vohra S, Marlin S, Ungar WJ, Offringa M. Guidelines for Reporting Outcomes in Trial Reports: The CONSORT-Outcomes 2022 Extension. JAMA. 2022 Dec 13;328(22):2252-2264. doi: 10.1001/jama.2022.21022. PMID: 36511921.
